# Supplementary material for: Phosphorous Magnetic Resonance Spectroscopy to Detect Regional Differences of Energy and Membrane Metabolism in Naïve Glioblastoma Multiforme
Source: Cancers (Basel). 2021 May 26;13(11):2598. doi: 10.3390/cancers13112598 (PMC8199363; doi:10.3390/cancers13112598)
Supplement: Supplementary file 1 [file cancers-13-02598-s001.zip › cancers-1107233-supplementary.pdf]

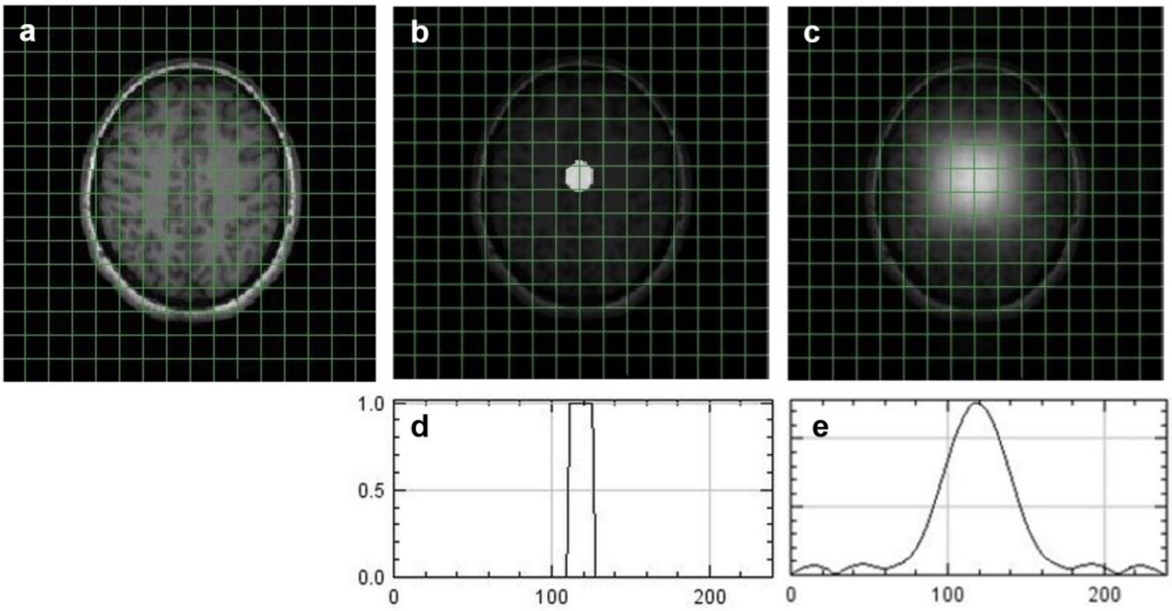

Figure S1. The figure indicates the effect of signal bleeding caused by a PSF obtained from data acquisition, with a matrix size of 8 × 8 × 8, extrapolated with zero-filling to 16 × 16 × 16 and Hamming filtering. Axial slice of the brain (a); the hyperintense spot in (b) indicates a lesion with the dimension of the voxel size, which is considerably blurred, extending into adjacent regions, as shown in (c). Intensity profiles for the original hyperintense spot (d) and the filtered image (e) are shown in the second row.
